# Methane Production Using Olive Tree Pruning Biomass Under H_2_O_2_ Pretreatment Enhanced with UV and Alkali

**DOI:** 10.3390/molecules30224379

**Published:** 2025-11-13

**Authors:** Fotini Antoniou, Ilias Apostolopoulos, Athanasia G. Tekerlekopoulou, Georgia Antonopoulou

**Affiliations:** 1 Department of Sustainable Agriculture, University of Patras, 2 Georgiou Seferi St., GR 30100 Agrinio, Greece; fayeantwniou97@gmail.com (F.A.); atekerle@upatras.gr (A.G.T.); 2 Institute of Chemical Engineering Sciences, Stadiou St., Platani, GR 26504 Patras, Greece; ilias_apost@hotmail.com

**Keywords:** olive tree pruning, UV, H_2_O_2_, alkali, pretreatment, biogas, anaerobic digestion, methane

## Abstract

Olive tree pruning (OTP), a widely available agricultural residue in Mediterranean countries, represents a promising lignocellulosic feedstock for anaerobic digestion. However, its recalcitrant structure limits its biodegradability and methane yields, necessitating effective pretreatment approaches. In this context, hydrogen peroxide in combination with ultraviolet (UV) radiation (UV/H_2_O_2_) at ambient temperature was used as a pretreatment method for enhancing methane production from OTP. Three concentrations of H_2_O_2_ (0, 1, and 3% *w*/*w*) alone or in combination with UV radiation, at different retention times (8, 14, and 20 h), were evaluated to enhance OTP depolymerization and methane generation. In addition, the combination of UV/H_2_O_2_ with alkali (UV/H_2_O_2_/NaOH) was compared with the typical alkaline pretreatment (NaOH) in terms of lignocellulosic biomass fractionation and biochemical methane potential (BMP). Results showed that increasing H_2_O_2_ concentration during UV/H_2_O_2_ pretreatment enhanced hemicellulose solubilization. Both NaOH and UV/H_2_O_2_/NaOH pretreatment promoted lignin reduction (37.3% and 37.8%), resulting in enhanced BMP values of 330.5 and 337.9 L CH_4_/kg TS, respectively. Considering operational energy requirements (heating at 80 °C and irradiance for 20 h) and methane energy recovery, net energy balances of 45.52 kJ and 66.65 kJ were obtained for NaOH and UV/H_2_O_2_/NaOH, respectively.

## 1. Introduction

The annual production of lignocellulosic biomass in the world is nearly 182 billion tons, yet only 3% of this biomass is utilized efficiently [1]. The olive sector, with a dedicated global surface area of 12 million hectares in over 40 countries [2], is poised to play a crucial role in the development of a bioeconomy, particularly in Mediterranean countries. During the cultivation of olive trees, 2.7 to 3.9 tons of biomass residues are produced per hectare, as a result of pruning (olive tree pruning—OTP). These residues are often left on-site or burned uncontrolled, leading to serious environmental pollution [3]. Due to its rich composition of carbohydrates, specifically cellulosic and hemicellulosic polymers (known as holocellulose), OTP serves as an ideal substrate for fermentative processes aimed at producing a wide range of bioproducts, in a biorefinery context [4].

Anaerobic digestion (AD) has been extensively studied for methane production from various lignocellulosic materials [5,6]. However, if lignocellulosic biomass is not treated properly, only 20–30% of its components, mainly the extractives and hemicellulose, are converted into methane during the AD process [5]. This limitation is primarily due to lignin, which provides mechanical and chemical stability [7] and is tightly bound to the holocellulose matrix. This binding makes the surface of cellulose less accessible to the enzymes needed for its depolymerization and conversion into sugars [8], which could be transformed into methane. In this context, applying an effective and specific pretreatment method to facilitate lignocellulose conversion towards methane is necessary [6].

Among the common pretreatment techniques, dilute acid treatment and steam explosion are still expensive and energy-intensive [9]. Additionally, they face technical challenges such as corrosion and the formation of inhibitory compounds that can affect the subsequent biological stages of the process [10]. To address the increasing market demand for sustainable solutions, the ongoing quest to reduce costs and scale up production has led to the development of new technologies. In this context, pretreatment with hydrogen peroxide (H_2_O_2_), which is typically carried out under mild conditions, generates free radicals (^•^OH and HOO^•^) and molecular oxygen [11], which improve the efficiency of enzymatic hydrolysis and enhance cellulose accessibility, resulting in higher sugar yields [12]. It was shown that alkaline hydrogen peroxide pretreatment (AHP) at a pH of 11.5 [13,14], or the combination of H_2_O_2_ with sodium hydroxide (NaOH) [15], enhances not only the solubilization of hemicellulose but also biomass delignification efficiency. The latter is significantly influenced by pretreatment pH, which facilitates or inhibits the formation of reactive oxygen radicals [16,17,18].

Another approach gaining attention is the use of H_2_O_2_ combined with ultraviolet (UV) irradiation (UV/H_2_O_2_). This method enhances the in-situ production of hydroxyl (^•^OH) radicals through rapid photochemical reactions [19]. However, there is still limited research on how UV/H_2_O_2_ pretreatment affects lignocellulosic materials. To date, only Yang et al. [20] have studied the combination of UV photocatalysis with AHP during the enzymatic hydrolysis of sisal waste. Their results showed that the combined method outperformed traditional AHP by shortening pretreatment time and enhancing enzymatic digestibility. Similarly, Hu et al. [21] studied the photocatalytic AHP method for corn stover and reported enhanced lignin removal and digestibility compared to the traditional AHP method. In general, different advanced oxidation processes (AOPs) have different mechanisms and present distinct practical strengths and limitations. For instance, AHP and UV-AHP are based on the oxidation and solubilization of lignin due to H_2_O_2_ [22]; UV/H_2_O_2_ increases ^•^OH radical formation and can markedly improve oxidant efficiency at ambient temperature, thereby reducing the need for increased temperatures compared to alkaline pretreatments [23]. Highly reactive radicals, which are often effective at delignification, are also produced during Photo-Fenton, but this method requires iron addition and pH control and sometimes forms different intermediate compounds that may influence downstream anaerobic activity [24]. Electrochemical generation of H_2_O_2_ on-site can minimize chemical transport and storage but shifts the operational burden to electrical energy and reactor complexity [24]. Comparative studies have shown that AOPs and photo-assisted systems can, in some cases, reduce the need for chemicals or heating energy. However, these benefits can be balanced with some limitations, such as reagent management and possible formation of inhibitory by-products, which strongly depend on the specific biomass type, oxidant concentration, reaction time, temperature, reactor mixing and biomass composition [25]. Given that these parameters affect delignification and carbohydrate preservation, the reported methane yields after AOP pretreatments vary widely across studies.

In the present study, H_2_O_2_ combined with UV radiation (UV/H_2_O_2_) at ambient temperature was applied as a pretreatment for improving the methane production of OTP, through biochemical methane potential (BMP). To the best of our knowledge, this is the first study to evaluate the effects of UV/H_2_O_2_ on the anaerobic digestibility and BMP of a lignocellulosic biomass feedstock. Three different concentrations of H_2_O_2_ (0, 1, and 3% *w*/*w*) were tested both independently and in combination with UV radiation, over varying retention times (8, 14, and 20 h), aiming at improving OTP solubilization, depolymerization as well as methane generation. Additionally, the combination of UV/H_2_O_2_ with sodium hydroxide (UV/H_2_O_2_/NaOH) was proposed as a hybrid approach that aims to combine the delignification efficiency of AOPs with the lignin-removal capacity of alkali, while keeping thermal energy requirements low, and compared to the typical traditional alkaline pretreatment using NaOH. Overall, this study not only provides comparative insights but also proposes a novel, energy-efficient oxidative pretreatment approach under mild operational conditions, which offers a promising alternative for enhancing biomass digestibility and increasing bioenergy production.

## 2. Results and Discussion

### 2.1. Effects of Pretreatments on OTP Characteristics

#### 2.1.1. Analysis of the Solid Fraction

The composition of OTP before pretreatment was: total solids (TS) = 92.0 ± 0.1 g/100 g, volatile solids (VS) = 91.9 ± 0.2 g/100 g TS, cellulose: 24.0 ± 0.5 g/100 g TS, hemicellulose: 15.9 ± 1.6 g/100 g TS, lignin: 41.3 ± 0.1 g/100 g TS, extractives: 16.9 ± 0.3 g/100 g TS, and proteins: 1.9 ± 0.0 g/100 g TS. The characteristics of the OTP used differed from those reported in other studies. For instance, in the study of Fonseca et al. [26], a holocellulose content in OTP exceeding 50% was reported, consisting of 36.5 ± 0.2% cellulose and 21.3 ± 0.4% hemicellulose, along with a relatively low lignin content of 24.1 ± 0.3%. Similarly, Santos et al. [27] observed a lower lignin content of 16.6%, but with holocellulose values comparable to those of the present study, i.e., 22.5% cellulose and 14.2% hemicellulose. The variability in the chemical composition of OTP can be attributed to its heterogeneous components, such as thin branches, woody tissues, and leaves, each with a distinct lignocellulosic composition. The final lignocellulose composition of OTP depends on the relative contribution of each component. Additionally, environmental factors and regional cultivation practices contribute to further variation. Despite these differences, OTP consistently shows a high holocellulosic content, rendering it an important source of bioenergy and biobased products, in the frame of a biorefinery approach [28].

**Figure 1:**
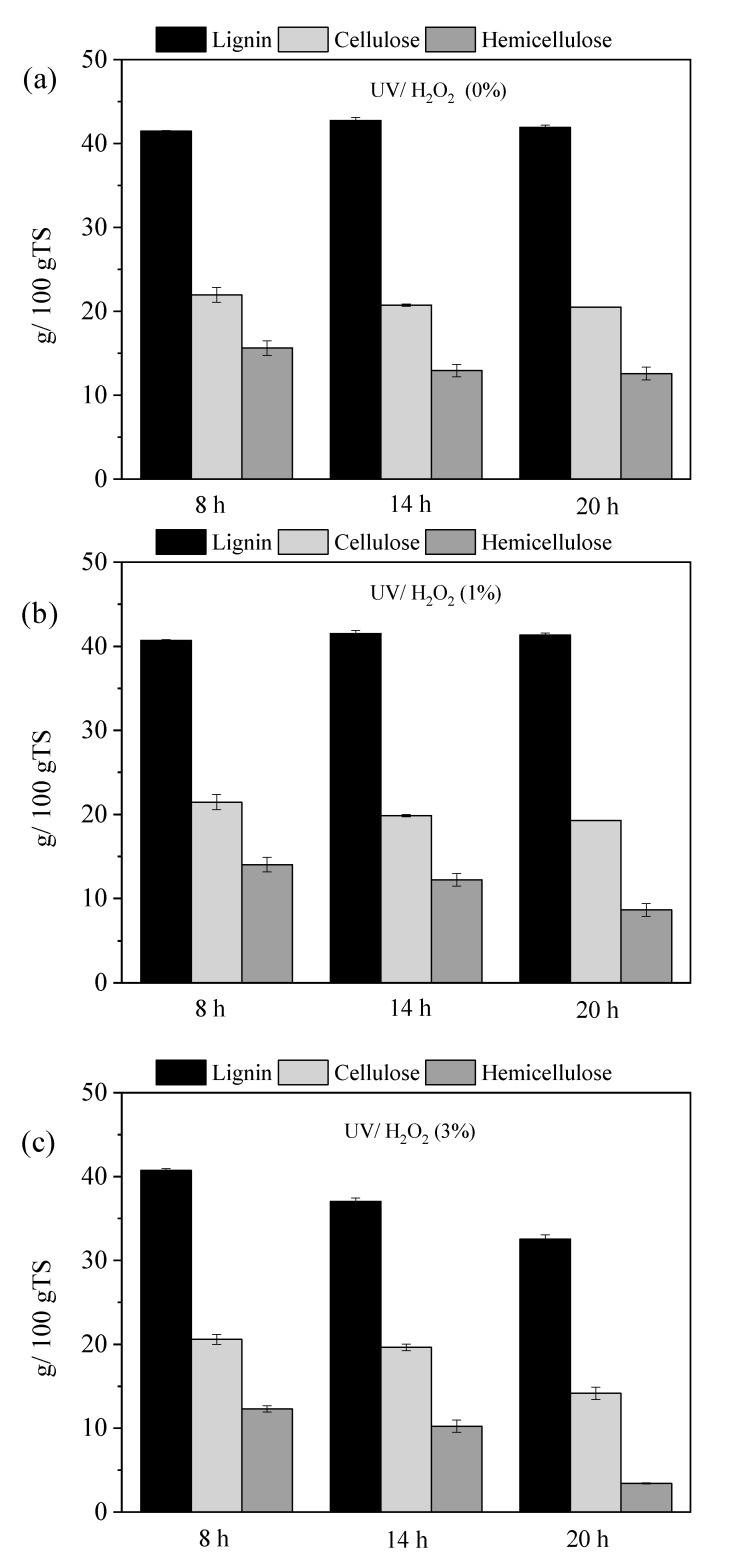
The effect of pretreatment duration (8, 14 and 20 h) on OTP lignocellulosic composition after treatment with UV/H_2_O_2_ at concentrations of (**a**) 0%, (**b**) 1% and (**c**) 3%.

Figure 1 illustrates the effect of pretreatment duration on OTP lignocellulosic composition after treatment with UV/H_2_O_2_ at various concentrations. It should be noted that the values are expressed per kg of initial TS (TS of non-pretreated OTP), taking into account the solid material recovery (MR) of biomass (Equation (2)), due to the mass loss during pretreatment. The lignocellulosic material (LM) after pretreatment was calculated according to Equation (3). Specifically, Figure 1a illustrates the lignocellulosic composition after pretreatment with 0% H_2_O_2_ (water only) for 8, 14, and 20 h, in combination with UV light. Figure 1b,c shows the lignocellulosic fractionation after pretreatment with 1% and 3% H_2_O_2_, respectively, for the same durations, also combined with UV light. The use of UV light without the chemical agent (0% H_2_O_2_) did not affect the lignin content, resulting only in a reduction in hemicellulose, which increased with the duration of the pretreatment (Figure 1a). At 8 h, hemicellulose reduction was only 1.7%, but this increased to 18.5% at 14 h and 20.8% at 20 h. In contrast, in the study of Mattonai et al. [29], degradation of lignin was observed when different lignocellulosic samples (fir, pine, chestnut, and oak wood) were pretreated with UV irradiation for 28 d at 60 °C and an irradiance of 800 W/m^2^. However, the longer pretreatment time and the higher UV intensity applied in that study might have affected the lignin content, which did not happen with the OTP in the current study.

From Figure 1b, it can be seen that the effect was more pronounced under the pretreatment with UV/H_2_O_2_ (1%), leading to a hemicellulose reduction of 11.7% at 8 h, 23.1% at 14 h, and 45.6% at 20 h. In general, when UV/H_2_O_2_ is applied, •OH is formed due to the direct photolysis of hydrogen peroxide according to reaction (R1) [30]. Then, the radicals •OH attack any organic compound R, causing chemical decomposition (R_oxid_), according to the reaction R2 [31]:

H_2_O_2_ + hv ⟶ 2 •OH    (R1)

R + •OH ⟶ R_oxid_         (R2)

Pretreatments lasting 14 and 20 h also had a slight impact on cellulose content, with reductions of 6.3% and 10.2%, respectively, but did not significantly affect lignin degradation. Lignin content was only influenced during the UV/H_2_O_2_ (3%) pretreatments, with reductions increasing with longer pretreatment times (Figure 1c).

Specifically, reductions in lignin were 1.4% at 8 h, 10.3% at 14 h, and 21.2% at 20 h, while cellulose content decreased by 8.4%, 10.7%, and 24.0%, and hemicellulose content decreased by 22.7%, 55.4%, and 78.5%, respectively.

**Figure 2:**
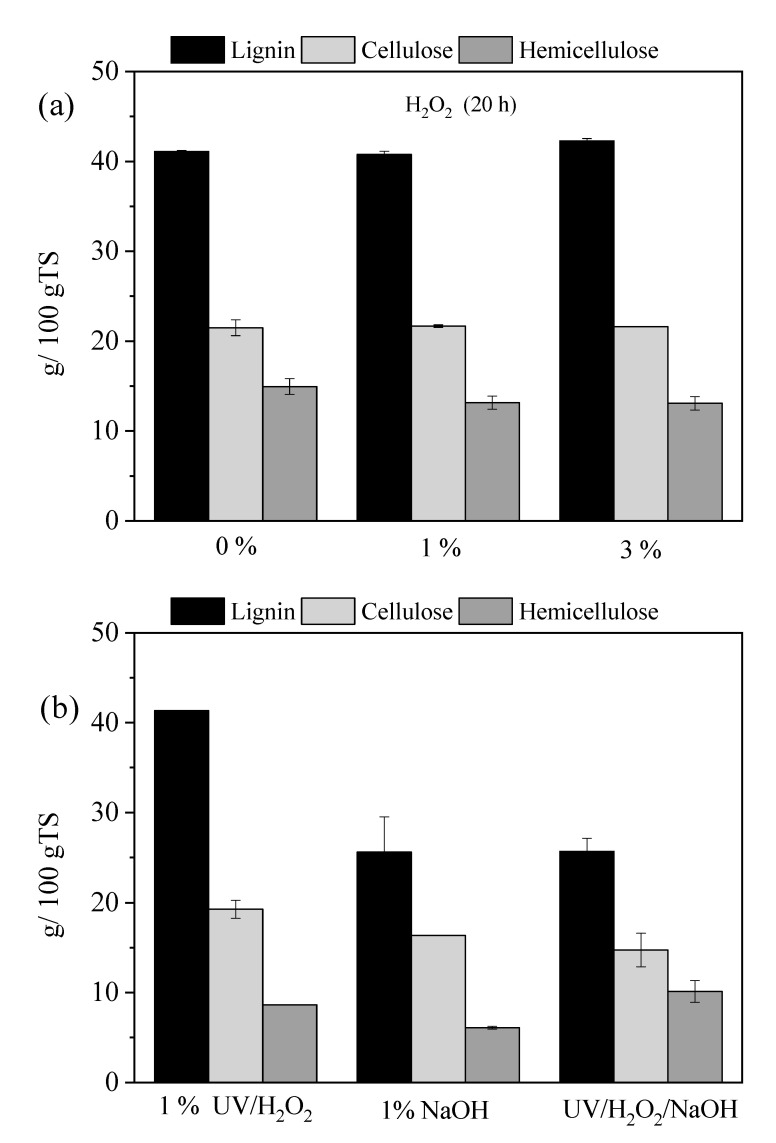
Lignocellulosic composition after pretreatment for 20 h with (**a**) H_2_O_2_ (0%, 1%, and 3%) and (**b**) NaOH (80° C), UV/H_2_O_2_/NaOH and 1% UV/H_2_O_2_.

After 20 h of UV pretreatment, comparison of the lignocellulose fractionation revealed that the concentration of H_2_O_2_ is essential, as it increases hemicellulose solubilization and influences lignin content. To evaluate the impact of H_2_O_2_ concentration during a pretreatment of 20 h, experiments were conducted using concentrations starting from 0% (water only), 1%, and 3% without UV radiation. The results on lignocellulose composition are illustrated in Figure 2a. Under these conditions, H_2_O_2_ alone did not significantly affect cellulose and lignin, but it caused a slight reduction in hemicellulose concentration, with reductions of 17.0% observed for both the 1% and 3% H_2_O_2_ concentrations. This finding aligns with previous studies, which reported that H_2_O_2_ applied alone on willow sawdust at a lower concentration (0.5% *v*/*v*) for 24 h did not significantly influence cellulose or lignin, but only hemicellulose content (5.2%) [15]. In contrast, when H_2_O_2_ at the same conditions was applied to date palm fibers, a high impact was observed in cellulose (19.6% reduction), hemicellulose (52.2% reduction), and lignin (15.1% reduction) content [32]. Different structural disruptions of both substrates can be attributed to their different lignocellulosic characteristics.

However, the combination of H_2_O_2_ pretreatment with an acid or alkali can significantly affect the pH, thereby promoting the delignification and/or depolymerization of the holocellulosic fraction. For instance, combining H_2_O_2_ with phosphoric acid was found to be effective for pretreating wheat straw, resulting in the removal of a substantial amount of lignin and disrupting the crystalline structure of cellulose [33]. A well-studied method is to add an alkali, such as sodium hydroxide (NaOH), or to adjust the pH to 11.5. Under these alkaline conditions, the delignification efficiency is enhanced by cleaving lignin side chains and breaking down lignin into low-molecular-weight compounds [13], thus facilitating the solubilization of hemicellulose while preserving cellulose [12].

To assess the influence of pH during UV/H_2_O_2_ pretreatment, a mixture containing 1% *w*/*v* NaOH and 1% *w*/*w* H_2_O_2_ was applied to the OTP and subjected to UV irradiation for 20 h (Figure 2b). For comparison, a conventional alkaline pretreatment using 1% *w*/*v* NaOH at 80 °C for 20 h was also conducted. The UV/H_2_O_2_/NaOH pretreatment resulted in a 37.8% reduction in lignin, along with 22.6% and 36.2% reductions in cellulose and hemicellulose. When compared to UV/H_2_O_2_ pretreatment alone, without NaOH addition (1% for 20 h), the UV/H_2_O_2_/NaOH demonstrated a notably higher delignification efficiency, since no significant lignin removal was observed under UV/H_2_O_2_. However, the reduction efficiency of hemicellulose was lower (45.6% under UV/H_2_O_2_ and 36.2% under UV/H_2_O_2_/NaOH), while cellulose loss was higher (11.6 and 22.6%, respectively). These results were consistent with those reported by Hu et al. [21], who observed a higher lignin removal efficiency of corn stover (from 73.3% to 86.1%) when AHP pretreatment was combined with UV light. Furthermore, increasing H_2_O_2_ concentration enhanced lignin removal efficiency (from 88.5 to 93.0%), even under constant reaction time and alkali concentrations. This indicates the crucial role of H_2_O_2_ concentration in lignocellulosic fractionation during AHP pretreatment, likely due to the generation of reactive oxygen species (e.g., hydroperoxyl radicals, HOO•). These radicals can cleave ether linkages and hinder lignin aggregation, thereby facilitating lignin solubilization [18,34]. In addition, under UV light, enhanced delignification can be observed due to the formation of •OH, which interacts with the chromogenic groups in the lignin structure, resulting in the formation of hydrophilic carboxyl and hydroxyl groups, which promote lignin solubilization [20].

As can be seen in Figure 2b, NaOH pretreatment at 80 °C for 20 h resulted in a reduction in lignin by 37.3%, along with decreases in cellulose and hemicellulose content by 18.6% and 61.6%, respectively. The delignification efficiency observed under these conditions was comparable to that achieved using the UV/H_2_O_2_/NaOH pretreatment. However, a direct comparison is not possible, as the NaOH pretreatment was conducted at a higher temperature (80 °C), which is considered optimal for alkali pretreatment [35]. The application of UV/H_2_O_2_/NaOH pretreatment at room temperature in OTP resulted in the same delignification efficiency as the optimum with NaOH. Alkaline conditions generally promote lignin breakdown [6], and this effect can be enhanced through the sequential use of other chemical agents, as in the current study. In addition, in Ben Atitallah’s [15] study, a two-stage pretreatment involving 0.5% (*w*/*v*) NaOH for 24 h at 80 °C followed by 0.5% (*v*/*v*) H_2_O_2_ for another 24 h resulted in the highest delignification efficiency of 38.3 ± 0.1%, even compared with the NaOH alone (0.5% (*w*/*v*) NaOH for 24 h at 80 °C).

Although the present study focuses on compositional fractionation and biodegradability, it is acknowledged that structural parameters such as cellulose crystallinity and specific surface area also influence digestibility. Techniques such as XRD (to assess crystallinity index) and BET (to quantify accessible surface area) are commonly used to describe these structural attributes and can provide complementary insight when interpreting pretreatment effects on microbial accessibility and methane yield.

#### 2.1.2. Analysis of the Liquid Fraction

The compounds produced during pretreatment significantly depend on the biomass used and the severity of the applied pretreatment method [36]. In general, under H_2_O_2_ or AHP pretreatments, no toxic compounds such as furans or acids are released [15]. Table 1 presents the concentrations of total carbohydrates, phenolic compounds, and chemical oxygen demand (COD) measured in the liquid fraction obtained after pretreatments. The data indicate that both carbohydrate and COD concentrations increased in proportion to the pretreatment severity, which is also aligned with the reductions in holocellulose and lignin, as illustrated in Figure 1 and Figure 2. For instance, the pretreatments using UV/H_2_O_2_/NaOH or NaOH alone, which resulted in significant hemicellulose and cellulose degradations, led to higher concentrations of carbohydrates, measured at 9.4 ± 0.6 g carbohydrates/100 g TS and 12.6 ± 0.9 g/100 g TS, respectively. Additionally, the solubilization of lignin contributed to even higher COD concentrations, measured as 41.3 ± 2.2 g/100 g TS and 45.2 ± 3.7 g/100 g TS, respectively.

Regarding the concentration of phenolic compounds, treatments using NaOH or H_2_O_2_ at concentrations of 0%, 1%, and 3%, or only UV light for durations of 8, 14, or 20 h (0% UV/H_2_O_2_) resulted in their production, which was proportional to the severity of the pretreatment. Thus, higher concentrations of the chemical agents and longer pretreatment times led to increased levels of phenolic compounds. Conversely, the combination of UV light with H_2_O_2_ at concentrations of 1% and 3% *w*/*w* resulted in a reduction in phenolic concentration when either the retention time or the H_2_O_2_ concentration increased. Specifically, at a UV/H_2_O_2_ concentration of 1% *w*/*w*, the phenolic compounds measured were 0.6 ± 0.1, 0.5 ± 0.0, and 0.4 ± 0.1 g/100 g TS after 8, 14, and 20 h of treatment, respectively, and at a UV/H_2_O_2_ concentration of 3% *w*/*w*, the respective values were 0.6 ± 0.0, 0.3 ± 0.0, and 0.3 ± 0.0 g/100g TS. This may be attributed to the non-selective nature of AOPs such as UV/H_2_O_2_, which are known to efficiently remove phenolic compounds [37]. Specifically, both UV/H_2_O_2_ and photo-Fenton processes are well-documented for their capacity to degrade/remove phenolic contaminants in wastewater. This efficiency is due to the generation of highly reactive hydroxyl radicals, which attack phenolic compounds, breaking them down into smaller, biodegradable intermediates or fully mineralizing them into CO_2_ and H_2_O [37]. In this case, degradation efficiency is influenced by many operational parameters, including H_2_O_2_ concentration, UV intensity, and retention duration. This means that the phenolic compounds, which were released during lignin degradation, were also degraded simultaneously. For this reason, AOPs such as UV/H_2_O_2_ and photo-Fenton can be considered as sustainable and efficient alternative treatment technologies for phenol-rich industrial effluents, offering significant reductions in the environmental impact of phenolic contaminants.

### 2.2. Effects of Pretreatments on the BMP of OTP

#### 2.2.1. BMP on the Whole Pretreatment Slurry

**Figure 3:**
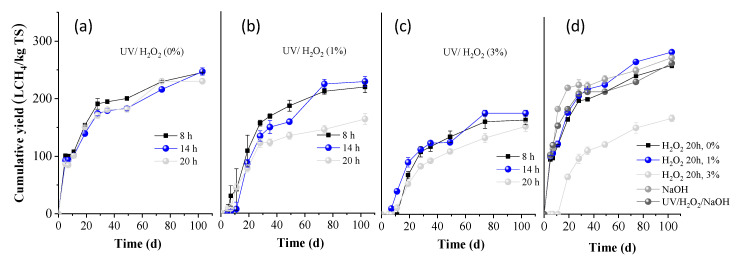
Cumulative methane yield of OTP pretreated with (**a**) UV/H_2_O_2_ (0%), (**b**) UV/H_2_O_2_ (1%), and (**c**) UV/H_2_O_2_ (3%) for 8, 14, and 20 h, respectively; (**d**) H_2_O_2_ (0, 1, and 3%), NaOH (80 °C), UV/H_2_O_2_/NaOH for 20 h.

BMP experiments were performed on the whole slurries obtained after all pretreatment conditions, and the results are presented in Figure 3 (expressed as L methane per kg of initial TS). The BMP of OTP without any treatment was measured as 264.7 ± 4.3 L CH_4_/kg TS (H_2_O_2_ 0%, 20 h, in Figure 3d). This value was slightly increased when treated for 20 h with H_2_O_2_ 1%, to 288.3 ± 2.5 L CH_4_/kg TS (Figure 3d). However, it was significantly decreased to 170.5 ± 5.8 L CH_4_/kg TS, when treated with H_2_O_2_ 3% for 20 h, indicating a high inhibition of methanogenesis.

From Figure 3a, it is evident that when OTP was pretreated with UV light alone, the BMP values remained similar for residence times of 8 and 14 h, being 245.6 ± 0.2 and 247.2 ± 6.6 L CH_4_/kg TS, respectively, (*p* > 0.05; the difference in the average values is not considered significant). However, a slight decrease was observed for the 20 h pretreatment (230.4 ± 4.1 L CH4/kg TS). When UV light was combined with H_2_O_2_ (1%), the BMP values were slightly lower, i.e., 219.1 ± 9.9 and 228.6± 8.8 L CH_4_/kg TS for 8 and 14 h, respectively. A significant reduction was observed after 20 h of pretreatment, decreasing to a value of 163.5 ± 9.5 L CH_4_/kg TS. For the treatment of UV/H_2_O_2_ (3%), the BMP values remained low, at 164.0 ± 8.3 and 165.9 ± 5.4 L CH_4_/kg TS for 8 and 14 h, respectively, and further decreased with increasing pretreatment time, to 152.8 ± 8.6 L CH_4_/kg TS at 20 h (*p* < 0.05; the difference in the average values is considered significant at all cases, compared to the OTP without any treatment). These results suggest that combining UV light with H_2_O_2_, particularly at higher concentrations and longer exposure times, methane production is significantly reduced. This could be attributed to the fact that reactive oxygen species, such as hydroxyl radicals, sometimes not only degrade organic material but may also produce other intermediate compounds, i.e., aldehydes, ketones, or carboxylic acids, which might inhibit methanogenic bacteria. Another explanation is that if H_2_O_2_ is not completely decomposed and a residual amount remains in the entire slurry, it can be harmful to anaerobic microbial communities, inhibiting methanogens. These arguments are also justified by the low BMP values of OTP pretreated with H_2_O_2_ 3% (without UV light), as presented above.

From Figure 3d, it is also obvious that alkali pretreatment alone positively affected the BMP (278.4 ± 3.7 L CH_4_/kg ΤS), while the combination of UV/H_2_O_2_/NaOH did not influence its value (268.5 ± 2.0 L CH_4_/kg ΤS). The slight increase in BMP under alkaline conditions can be attributed to the lignin reduction that occurred (Figure 2b). Under alkaline conditions, cleavage of lignin-carbohydrate linkages occurs, resulting in a smoother structure and consequently enhancing biodegradability during AD [6]. However, the increase in BMP was not as significant as expected, given the substantial lignin reduction observed. A possible explanation could be the high concentration of phenolics, which were produced (6.2 ± 0.5 g/100 g TS) that might cause a partial inhibition of methanogens.

#### 2.2.2. BMP on the Liquid and Solid Fractions Remained After Pretreatment

**Figure 4:**
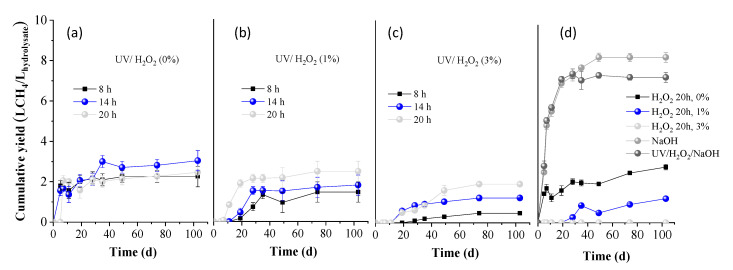
Cumulative methane yield of the liquid fraction obtained after OTP pretreatment with (**a**) UV/H_2_O_2_ (0%), (**b**) UV/H_2_O_2_ (1%), and (**c**) UV/H_2_O_2_ (3%) for 8, 14, and 20 h, respectively; (**d**) H_2_O_2_ (0, 1, and 3%), NaOH (80 °C), UV/H_2_O_2_/NaOH for 20 h.

In Figure 4, the BMP of the liquid fractions obtained after pretreatments is presented, expressed as L CH_4_ per L of hydrolysate used. The BMP of 0% H_2_O_2_ (only water) for 20 h (Figure 4d) was calculated as 2.8 ± 0.1 L/L, indicating limited solubilization during the 20 h hydrolysis of OTP with water. The BMP did not remarkably change when OTP was hydrolyzed with water under UV light for 8, 14, and 20 h, yielding 2.2 ± 0.5, 3.0 ± 0.5, and 2.5 ± 0.3 L CH_4_/L hydrolysate, respectively. However, these values decreased under the combination of 1 or 3% H_2_O_2_ with UV light, indicating a slight inhibition, due to the oxidative conditions caused and the possible formation of inhibitory byproducts. While UV/H_2_O_2_ can enhance solubilization, as observed by the carbohydrate concentration values in Table 1, excessive oxidation seems to counteract this benefit. On the other hand, the use of 1% H_2_O_2_ for 20 h without UV exposure resulted in a reduced BMP to 1.2 ± 0.2 L CH_4_/L hydrolysate, indicating a negative impact on methane production. Moreover, the use of 3% H_2_O_2_ led to complete inhibition of the BMP, likely due to excessive oxidation conditions, causing oxidative stress to AD performance. Finally, pretreatment with NaOH or UV/H_2_O_2_/NaOH led to enhanced BMP values of 8.4 ± 0.7 and 7.5 ± 0.9 L CH_4_/L hydrolysate, respectively. This indicates that the presence of high concentrations of phenolic compounds did not hinder anaerobic microbial activity, allowing efficient degradation of the abundant carbohydrates in the hydrolysate. Comparable results (9.4 ± 0.9 L CH_4_/L hydrolysate) were reported in the study of Antonopoulou et al. [35], where grass lawn pretreated with 1% NaOH yielded similarly high BMP. This was attributed to the high organic and sugar content in the hydrolysate, released because of hemicellulose solubilization.

**Figure 5:**
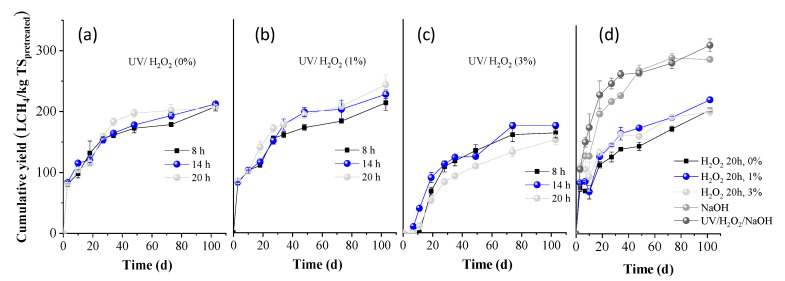
Cumulative methane yield of the solid fraction obtained after OTP pretreatment with (**a**) UV/H_2_O_2_ (0%), (**b**) UV/H_2_O_2_ (1%), and (**c**) UV/H_2_O_2_ (3%) for 8, 14, and 20 h, respectively; (**d**) H_2_O_2_ (0, 1, and 3%), NaOH (80 °C), UV/H_2_O_2_/NaOH for 20 h.

In Figure 5, the BMP of the solid fractions obtained after pretreatments is presented, expressed as L of methane per kg TS of pretreated solids. The BMP of 0% H_2_O_2_ (only water) for 20 h (Figure 5d) was calculated as 208.3 ± 3.1 L/kg TS, and was not remarkably changed when OTP was treated with water under UV light for 8, 14, and 20 h (Figure 5a), which was 208.1 ± 0.7, 212.5 ± 3.1, and 213.4 ± 12.4 L CH_4_/kg TS pretreated solids, respectively. In contrast, these values increased under the combination of 1 or 3% H_2_O_2_ with UV light, and the higher the pretreatment time, the higher the BMP (213.4 ± 12.4, 227.5 ± 6.9 and 243.1 ± 15.9 L CH_4_/kg TS at 8, 14 and 20 h under 1% H_2_O_2_ and 217.8 ± 14.1, 234.2 ± 3.3 and 241.0 ± 0.8 L CH_4_/kg TS under 3% H_2_O_2_, respectively). This can be attributed to the effect of pretreatments on the lignocellulosics. Under NaOH or UV/H_2_O_2_/NaOH, in which lignin removal was observed, the BMP was higher, reaching up to 309.7 ± 10.5 and 293.3 ± 2.4 L CH_4_/kg TS, respectively.

#### 2.2.3. Comparison of the BMPs of the Whole Slurries with the Separated Fractions

For comparing the BMPs, all the results are expressed as per kg TS of initial biomass (untreated OTP), i.e., as L CH_4_/kg TSin. Thus, assuming that no liquid was lost during pretreatment and that the volume of the hydrolysate was stable, the BMP of the hydrolysate can be calculated in the respective units. In addition, taking into account the MR (Equation (2)), the BMP of the solid fractions was calculated using Equation (4). The results regarding the BMPs of all fractions (whole biomass, solid, and liquid fractions) are presented in Table 2.

Regarding the solids, higher BMP yields were observed for pretreatment with 1% UV/H_2_O_2_, at all pretreatment times, and the higher the pretreatment time, the higher the BMP (i.e., 190.7 ± 11.1, 196.4 ± 6.0 and 200.4 ± 13.2 L CH_4_/kg TSin for 8, 14 and 20 h, respectively). Pretreatment with 1% H_2_O_2_ without UV also led to a high BMP of 202.3 ± 3.5 L CH_4_/kg TSin, whereas treatment with NaOH led to the lowest BMP value of 162.9 ± 1.5 L CH_4_/kg TSin, due to the lowest value of MR, which was observed under these pretreatment conditions (52.6%). Indeed, as seen in Figure 2b, NaOH pretreatment led to a high solubilization of lignin, cellulose, and hemicellulose.

When comparing the BMPs of both fractions (sum of the BMP of liquid and solid fractions in Table 2), expressed in L CH_4_/kg TSin with that of the whole slurry at each pretreatment, it is clear that direct AD without separation of the pretreated biomass was favored only in the cases of 0 and 1% H_2_O_2_ and 0% UV/H_2_O_2_ for 8 h. In contrast, similar BMPs were observed for 0% UV/H_2_O_2_ for 14 or 20 h and 1% UV/H_2_O_2_ for 8 or 14 h, respectively, which favors the process economy. The use of the whole slurry reduces process costs by eliminating the need for separation. Treatment with 3% H_2_O_2_ for 20 h led to the lowest BMP either by using the whole slurry or the separated fractions, due to the inhibitory effect of H_2_O_2_ on methanogenic bacteria. On the other side, treatment with 1% UV/H_2_O_2_ for 20 h, 3% UV/H_2_O_2_ at all retention times, and NaOH or UV/H_2_O_2_/NaOH resulted in higher BMP values after separation of both fractions compared to the whole slurry. Especially for the NaOH and UV/H_2_O_2_/NaOH pretreatments, the maximum BMP values of 330.5 and 337.9 L CH_4_/kg TSin were achieved when adding both fractions.

Improved BMPs ranging from 85 to 156% were reported by Alexandropoulou et al. [5], who applied different H_2_O_2_/NaOH approaches (without UV) in willow sawdust biomass. In that study, the highest BMP was 267.2 ± 18.0 L/kg TS, which was achieved when a mixture of NaOH and H_2_O_2_ at a 1:1 ratio was applied before the BMPs. In a study conducted by Nitsos et al. [38], various pretreatment methods, including hydrothermal, dilute acid, and steam explosion, were applied to OTP. BMP experiments were then conducted either with or without enzyme addition. The BMP of untreated OTP was 56.8 ± 0.3 L CH_4_/kg VS and increased to 315.4 ± 0.0 L CH_4_/kg VS when steam explosion was applied. In contrast, the hydrothermal and diluted acid pretreatments resulted in lower BMP values of 93.1 ± 1.6 and 84.8 ± 0.0 L CH_4_/kg VS, respectively. The different methane yields reported in the literature, even in AHP (H_2_O_2_/NaOH) pretreatment, can be attributed to several factors, linked to both pretreatment conditions and biomass characteristics. Firstly, operational parameters such as H_2_O_2_ or NaOH concentrations, applied temperature, solids loading (solids-to-liquid ratio), and pretreatment time play a crucial role in lignin solubilization and cellulose accessibility [5]. Even small variations in these parameters can significantly alter delignification efficiency, carbohydrate solubilization, and product formation [15]. Secondly, biomass structure and chemical composition significantly influence pretreatment efficiency [5]. The relative composition of lignocellulose and extractives, as well as lignin structure and crystallinity of cellulose, differ among lignocellulosic biomasses [5]. Moreover, reactor configurations and mass transfer conditions (static or stirred) during pretreatment, which influence the process reaction kinetics, vary among studies. Finally, it is important to highlight that the current study introduced UV irradiation in combination with H_2_O_2_ and NaOH. This hybrid process has not been previously reported in the literature for any type of biomass. Considering all the above aspects, and particularly the synergistic effect of the UV in AOPs, provides a possible explanation for the different biodegradability and methane yields observed in this study compared with conventional H_2_O_2_/NaOH pretreatments reported in the literature.

#### 2.2.4. Energy Balances

Based on the results of the current study, treating OTP with NaOH (20 h at 80 °C) or combining NaOH with UV/H_2_O_2_ (20 h at 25 °C) seems to be the most promising approaches. It is important to separate both pretreatment fractions before AD, and each fraction should undergo AD in separate bioreactors. Comparing both processes in terms of their cost, the capital cost will be similar, as the cost associated with all tanks (the pretreatment tank and two anaerobic digesters) is identical for both processes. The main difference lies in the operational cost of the pretreatment. For NaOH pretreatment, the energy required to heat the suspension from 25 °C to 80 °C, expressed in kWh (dividing by 3600), can be estimated as:

(1) $$\mathrm{Heat}\;\mathrm{energy}\;\mathrm{requirement}\;=\;\frac{m\;\times \;Cp\;\times \;(T_{final}\;-\;T_{initial})}{3600}$$

where m is the mass of water and substrate in kg; Cp is the water specific heat value (4.18 kJ/kg °C); T_initial_ and T_final_ are the initial and final temperatures, assumed as 25 °C and 80 °C, respectively.

The heat energy requirement for a suspension of 5% TS was estimated as 22.99 kJ, which is equivalent to 0.0064 kWh. For a duration of 20 h, this corresponds to an energy requirement of 0.00032 kW. The reaction time of 20 h refers to the duration of pretreatment, not to the heating phase, which is assumed to occur once before maintaining the target temperature. The heating energy was calculated for the whole suspension (mass of water and substrate in kg) based on an initial solids’ concentration of 5% TS. During the pretreatment experiments, solid material solubilized remained in the suspension and was not removed before heating; therefore, the total mass that was heated did not change, and the calculated energy required to raise the suspension temperature is effectively independent of MR. By contrast, if an alternative scenario were used, e.g., remove and separately treat the liquid and the solids, the energy demand per unit of retained solid would change, and MR would need to be explicitly included in Equation (1).

To estimate the energy requirements for applying UV irradiation to the same suspension for 20 h, a 15 W lamp will theoretically consume 0.3 kWh. However, this value reflects the total energy consumption of the lamp, regardless of the sample volume. According to the Philips TUV 15W T8 lamp specifications, the irradiance is 48 μW/cm^2^. Given that the total surface area of the samples was 94.97 cm^2^ (corresponding to 4 cylindrical vials with a diameter of 5.5 cm, with 1.25 g TS each), the actual power delivered to the samples was equal to 4.55 mW or 0.00455 W or 0.000091 kWh. This is significantly lower than the energy required for the alkaline pretreatment process. For calculating the energy in kJ, the power (W) should be multiplied by the pretreatment time (20 h or 72,000 s), and the consumed energy during the pretreatment is 327.6 J or 0.328 kJ.

For calculating the energy gained in the form of methane upon both pretreatment options, its volumetric energy density should be considered (39.64 kJ/L CH_4_). For the NaOH pretreatment, from a suspension of 5% TS, 65.51 kJ was recovered, while combining NaOH with UV/H_2_O_2_, 66.97 kJ was produced. Considering the energy for heating a suspension of 5%TS at 80 °C for 20 h (22.99 kJ) and irradiance for 20 h (0.328 kJ), the net energy can be calculated (45.52 kJ for NaOH and 66.65 kJ for UV/H_2_O_2_/NaOH) and is presented in Table 3. It should be pointed out that in the current preliminary analysis, the cost of chemicals or other operational costs was not considered. Based on the above, the UV/H_2_O_2_/NaOH pretreatment seems to be a promising, energy-efficient method for producing enhanced methane from OTP.

## 3. Materials and Methods

### 3.1. Pretreatments of OTP

The OTP was chopped, milled, and then sieved to create a powder < 0.7 mm, which was subsequently air-dried at ambient temperature. For all pretreatment methods used, the solid loading was set at 5% *w*/*v* (5 g of TS of OTP in 100 mL of water). A UV lamp (TUV T8, Philips, The Netherlands, 15 W) emitting UV irradiation at 245 nm was utilized in the experiments. For UV/H_2_O_2_ pretreatment, three different concentrations of H_2_O_2_ were tested: 0%, 1%, and 3% *w*/*w*, in combination with UV irradiation at different retention times (8, 14, and 20 h). The samples were placed into four cylindrical vials made of quartz, each containing 25 mL of liquid and 1.25 g TS of OTP powder. Additionally, pretreatments with 0% (only water), 1% and 3% *w*/*w* H_2_O_2_ were applied to OTP for 20 h without UV irradiation. All the vials were placed in magnetic stirrers to ensure homogeneous contact between the solid and liquid phases, at a constant temperature of 25 °C. The combination of UV treatment with 1% *w*/*w* H_2_O_2_ was also tested for 20 h, alongside 1% *w*/*v* NaOH (referred to as UV/H_2_O_2_/NaOH), and this was compared to the typical-conventional NaOH pretreatment (1% *w*/*v* NaOH for 20 h at 80° C, placed in a rotating water bath). All pretreatment methods are presented in Table 4. After the pretreatment process, the whole slurry was separated through vacuum filtration using a 0.7 μm pore size filter, resulting in a liquid and a solid fraction. The solid fraction was thoroughly washed with distilled water until neutral pH was reached (pH ≈ 7) to eliminate any residual NaOH or H_2_O_2_. A detailed physicochemical and structural characterization was conducted on both fractions, based on the methodology presented by Antonopoulou et al. [35]. The free-extracted biomass was analyzed for the structural lignocellulosic content of untreated OTP. Both fractions and the whole pretreatment slurry (before separation) were tested for methane production through batch BMP tests.

### 3.2. BMP Tests

BMP tests were conducted in duplicate using the whole pretreatment slurry, as well as the liquid and solid fractions from all the pretreatment methods tested. The BMP tests followed the protocol reported in Antonopoulou et al. [35], utilizing anaerobic sludge as the microbial inoculum. The characteristics of the inoculum were as follows: pH: 6.9 ± 0.2, total suspended solids (TSS): 36.0 ± 0.8 g/L, volatile suspended solids (VSS): 19.4 ± 0.3 g/L, and soluble COD: 0.1 ± 0.0 g/L. Biogas production was monitored over time using a gas-tight syringe.

### 3.3. Analytical Methods

TS, VS, TSS, VSS, Total Kjeldahl Nitrogen (TKN), and soluble COD were analyzed following the Standard Methods reported by APHA [39]. The concentration of carbohydrates was measured according to the method described by Joseffson et al. [40] while phenolic compounds were assessed using the Folin–Ciocalteu method [41]. The protocols established by the National Renewable Energy Laboratory (NREL) and reported by Sluiter et al. [42,43] were used to determine the extractives and lignocellulosic material before and after pretreatments. The methane content of the produced biogas was determined using a gas chromatograph (SRI 8610C MG#1) equipped with a thermal conductivity detector (TCD) and a packed column. Helium was used as the carrier gas. The injector, column, and detector temperatures were set at 90 °C, 35 °C, and 100 °C, respectively. For calculation of the protein content in OTP, the TKN value was multiplied by a factor of 6.25.

### 3.4. Calculations

To calculate the LM (lignin, cellulose and hemicellulose) after pretreatment, the MR was taken into account, as presented in Equations (2) and (3):

(2) $$MR=\frac{{TS}_{pretreated}\;}{TS_{in}\;}\;\;\;\;\;\;\;\;$$

(3) $$LM\left(\frac{g}{100g\;{TS}_{in}}\right)=LM\left(\frac{g}{100g\;{TS}_{pretreated}}\right)\times MR\left(\frac{{TS}_{pretreated}}{TS_{in}}\right)$$

TS_in_ and TS_pretreated_ represent the TS content before and after pretreatment (in g).

To calculate the BMP of the solid fraction that remained after pretreatment, Equation (4) was used:

(4) $$BMP\left(\frac{L}{kg\;{TS}_{in}}\right)=BMP\left(\frac{L}{kg\;{TS}_{pretreated}}\right)\times MR\left(\frac{{TS}_{pretreated}}{TS_{in}}\right)$$

### 3.5. Statistical Analysis

A two-sample *t*-test with a threshold *p*-value of 0.05 was applied to statistically analyze the effect of pretreatment on the BMP of OTP.

## 4. Conclusions

This study demonstrated that UV/H_2_O_2_ pretreatment, particularly when combined with NaOH, is an effective method for enhancing methane production from olive tree pruning (OTP). Increasing H_2_O_2_ concentrations under UV exposure improved hemicellulose solubilization, while both NaOH and UV/H_2_O_2_/NaOH pretreatments significantly reduced lignin content by 37.3% and 37.8%, respectively. These structural modifications led to higher biochemical methane potentials (BMPs) of 330.5 and 337.9 L CH_4_/kg TS. Importantly, BMP values were maximized when the solid and liquid fractions were separated and digested in individual anaerobic reactors. Energy balance analysis confirmed that the net energy gain was substantial, with UV/H_2_O_2_/NaOH pretreatment yielding a higher recovery (66.65 kJ) than NaOH alone (45.52 kJ), due to its lower energy input and higher methane output. Overall, the UV/H_2_O_2_/NaOH pretreatment approach offers a promising, energy-efficient strategy for valorizing lignocellulosic waste like OTP through enhanced methane production.

## Figures and Tables

**Table 1:** The concentrations of total carbohydrates, phenolic compounds, and chemical oxygen demand (COD) measured in the liquid fraction obtained after pretreatments.

Pretreatment	Concentration (% *w*/*w*)	Time (h)	Carbohydrates (g/100 g TS)	Phenolics (g/100 g TS)	COD (g/100 g TS)
UV/H_2_O_2_	0	8	4.6 ± 0.2	1.2 ± 0.2	13.2 ± 0.9
		14	5.3 ± 0.5	1.5 ± 0.2	14.1 ± 1.1
		20	5.6 ± 0.3	1.5 ± 0.4	15.4 ± 1.2
	1	8	5.5 ± 0.4	0.6 ± 0.1	20.1 ± 0.9
		14	6.6 ± 0.4	0.5 ± 0.0	24.4 ± 1.2
		20	7.1 ± 0.8	0.4 ± 0.1	24.7 ± 1.2
	3	8	7.4 ± 0.9	0.6 ± 0.0	35.5 ± 0.6
		14	7.6 ± 0.2	0.3 ± 0.0	39.2 ± 2.2
		20	8.9 ± 0.2	0.3 ± 0.0	42.4 ± 1.1
H_2_O_2_	0	20	3.4 ± 0.5	0.9 ± 0.0	9.8 ± 0.9
	1	20	4.5 ± 0.3	0.7 ± 0.0	17.0 ± 1.2
	3	20	6.8 ± 0.9	1.1 ± 0.12	21.7 ± 1.3
UV/H_2_O_2_/NaOH	1, 1	20	9.4 ± 0.6	2.0 ± 0.2	41.3 ± 2.2
NaOH	1	20	12.6 ± 0.9	6.2 ± 0.5	45.2 ± 3.7

**Table 2:** BMP for whole pretreatment slurries and separated fractions obtained after all pretreatment methods.

Pretreatment	Concentration (% *w*/*w*)	Time (h)	Liquids (L CH_4_/kg TSin)	Solids (L CH_4_/kg TSin)	Whole Slurries (L CH_4_/kg TSin)
UV/H_2_O_2_	0	8	45.0 ± 5.2	185.9 ± 6.3	245.6 ± 0.2
		14	60.8 ± 5.5	189.5 ± 2.8	247.2 ± 6.6
		20	49.6 ± 3.3	184.1 ± 5.3	230.4 ± 4.1
	1	8	29.2 ± 3.4	190.7 ± 11.1	219.1 ± 9.9
		14	36.1 ± 4.5	196.4 ± 6.0	228.6 ± 8.8
		20	49.8 ± 3.9	200.4 ± 13.2	163.5 ± 9.5
	3	8	9.4 ± 0.9	188.8 ± 12.2	164.0 ± 8.3
		14	24.6 ± 2.3	193.7 ± 2.8	175.9 ± 5.4
		20	38.3 ± 3.7	188.7 ± 0.6	152.8 ± 8.6
H_2_O_2_	0	20	56.3 ± 1.3	186.4 ± 2.7	264.7 ± 4.3
	1	20	24.3 ± 0.3	202.3 ± 3.5	288.3 ± 2.5
	3	20	0.0 ± 0.0	184.0 ± 5.3	170.5 ± 5.8
UV/H_2_O_2_/NaOH	1, 1	20	147.3 ± 9.9	190.63 ± 1.6	268.5 ± 2.0
NaOH	1	20	167.6 ± 5.7	162.96 ± 5.5	278.4 ± 3.7

**Table 3:** Energy analysis for UV/H_2_O_2_/NaOH and NaOH pretreatment of OTP, where methane was produced after separating into both liquid and solid fractions. The analysis was based on the current pretreatment conditions (5% TS).

Pretreatment Conditions	Energy Gained from CH_4_ (kJ)	Energy Supply for Pretreatment (kJ)	Net Energy (kJ)
UV/H_2_O_2_/NaOH	65.51	22.99	42.52
NaOH	66.98	0.328	66.65

**Table 4:** Pretreatment conditions tested in the current study.

Symbol	Concentration (% *w*/*w*)	Temperature (°C)	Time (h)
UV/H_2_O_2_	0, 1, 3	25	8, 14, 20
H_2_O_2_	0, 1, 3	25	20
UV/H_2_O_2_/NaOH	1 (H_2_O_2_), 1 (NaOH)	25	20
NaOH	1	80	20

## Data Availability

Data will be made available on request.
